# Persistence of Antibodies Against Spike Glycoprotein of SARS-CoV-2 in Healthcare Workers Post Double Dose of BBV-152 and AZD1222 Vaccines

**DOI:** 10.3389/fmed.2021.778129

**Published:** 2021-12-22

**Authors:** Hari Ram Choudhary, Debaprasad Parai, Girish Chandra Dash, Jaya Singh Kshatri, Narayan Mishra, Prasanta Kumar Choudhary, Dipti Pattnaik, Kumudini Panigrahi, Susmita Behera, Nihar Ranjan Sahoo, Sreeparna Podder, Adyasha Mishra, Sunil Kumar Raghav, Sanjeeb Kumar Mishra, Subrat Kumar Pradhan, Subrat Kumar Sahoo, Matrujyoti Pattnaik, Usha Kiran Rout, Rashmi Ranjan Nanda, Nityananda Mondal, Srikanta Kanungo, Subrata Kumar Palo, Debdutta Bhattacharya, Sanghamitra Pati

**Affiliations:** ^1^Department of Microbiology, ICMR - Regional Medical Research Centre,Department of Health Research,Ministry of Health & Family Welfare, Government of India, Bhubaneswar, India; ^2^The Chest Clinic, Brahmapur, India; ^3^Department of Microbiology, Kalinga Institute of Medical Sciences, Bhubaneswar, India; ^4^Maharaja Krushna Chandra Gajapati College & Hospital, Berhampur, India; ^5^DBT – Institute of Life Sciences, Bhubaneswar, India; ^6^Department of Community Medicine, Veer Surendra Sai Institute of Medical Sciences and Research, Burla, India

**Keywords:** persistence, SARS-CoV-2, spike glycoprotein, healthcare worker, BBV-152 and AZD1222

## Abstract

**Purpose:** We investigated the persistence of the vaccine-induced immunoglobulin G (IgG) antibodies against severe acute respiratory syndrome coronavirus 2 (SARS-CoV-2) among healthcare workers (HCWs) in Odisha who received a complete dose of either Covaxin or Covishield vaccine.

**Methods:** This 24-week longitudinal cohort study was conducted from January to July 2021 with participants from 6 healthcare and research facilities of Odisha to understand the dynamicity of the vaccine-induced IgG antibodies against SARS-CoV-2 after the complete dose of vaccines.

**Results:** Serum samples were collected from 614 participants during each follow-up and were tested in two chemiluminescent microparticle immunoassay (CLIA)-based platforms to detect SARS-CoV-2 antibodies both qualitatively and quantitatively. Among these participants, 308 (50.2%) participants were Covishield recipients and the rest 306 (49.8%) participants took Covaxin. A total of 81 breakthrough cases were recorded and the rest 533 HCWs without any history of postvaccination infection showed significant antibody waning either from T3 (Covaxin recipient) or T4 (Covishield recipient). The production of vaccine-induced IgG antibodies is significantly higher (*p* < 0.001) in Covishield compared with Covaxin. Covishield recipients produced higher median anti-S IgG titer than Covaxin. No statistically significant differences in antibody titers were observed based on age, gender, comorbidities, and blood groups.

**Conclusion:** This 6-month follow-up study documents a 2-fold and 4-fold decrease in spike antibody titer among Covishield and Covaxin recipients, respectively. The clinical implications of antibody waning after vaccination are not well understood. It also highlights the need for further data to understand the long-term persistence of vaccine-induced antibody and threshold antibody titer required for protection against reinfection.

## Introduction

The unprecedented effort in vaccine development during this ongoing coronavirus diseae 2019 (COVID-19) pandemic has led to emergency approval of the various severe acute respiratory syndrome coronavirus 2 (SARS-CoV-2) vaccines around the world (1). The pandemic which continues to unfold in various waves has impacted health, by challenging the mortality rate for individuals with preexisting health conditions in the older and younger age groups (2). While the scientific community was trying to curb the impact of the first wave of COVID-19, the entire world got hit by another wave (3). Globally, 233.14 million people were infected with infection of COVID-19 and about 4.77 million COVID-19 deaths had been reported as of September 30, 2021 (4). India, Brazil, and the United States of America accounted for most of the cases worldwide wherein, India reported almost 33.74 million cases with 0.45 million deaths (5).

Spike (S) glycoprotein of coronavirus is the main target for the design of the leading SARS-CoV-2 vaccines such as Moderna's mRNA-1273, Pfizer's BNT162b2, and ChAdOx1 nCoV-19/AZD1222 (6). The vaccines have been rolled out worldwide to gain control of COVID-19 and reduce mortality and morbidity due to the virus (7). The emergency use of the two indigenous COVID-19 vaccines (BBV-152/Covaxin and AZD1222/Covishield) was authorized in India and subsequently, the largest vaccination drive of the world has been undertaken by the Ministry of Health & Family Welfare, Government of India (8). The drive was conducted across the country in a phased manner started from January 16, 2021. BBV152 (Bharat Biotech, Hyderabad, India) is a whole-virion ß-propiolactone-inactivated SARS-CoV-2 vaccine. Efficacy rate of Covaxin was recorded as 77.8% against symptomatic COVID-19 disease after 14 days of two doses (9). AZD1222 was co-invented by the University of Oxford and its spin-out company, Vaccitech. It uses a replication-deficient chimpanzee viral vector based on a weakened version of a common cold virus (adenovirus) that causes infections in chimpanzees and contains the genetic material of the SARS-CoV-2 virus spike protein. The AstraZeneca US Phase III trial of Covishield demonstrated statistically significant vaccine efficacy of 79% at preventing symptomatic COVID-19 and 100% efficacy at preventing severe disease and hospitalization (10). Initially, both the vaccines were being administered with two doses in 1:1 ratio at a gap of 28 days but later the duration was increased from 28 days to 84 days for Covishield vaccine in India. India has been able to administer vaccines to 919.7 million people out of which 252.7 million have been completely vaccinated with the BBV-152 or AZD1222 vaccine. Odisha has successfully administered vaccines to about 31.9 million people out of which about 9 million people have been completely vaccinated till September end (11).

The study of antibody magnitude and persistence is crucial to define their role in antigen clearance and protective capacity against future infection. Studies have found that antibodies developed against SARS-CoV-2 following natural infection and/or vaccination can be faded off with time (12, 13). In view of the increasing incidence of newly emergent SARS-CoV-2 variants, long-term studies are needed to understand the same. In this study, we have analyzed the persistence of antibodies among the BBV-152 and AZD1222 recipients in Odisha.

## Methods

### Study Settings

This longitudinal cohort study was conducted from January 16 to July 31, 2021 with participants from 6 different private/government healthcare and research facilities from 3 districts of Odisha, India. A total of 3 milliliters of venous blood samples were collected from 614 vaccinated adult individuals (aged 18 years and above) and sent for testing at Cobas Laboratoryof ICMR—Regional Medical Research Center, Bhubaneswar maintaining the cold chain.

### Study Design

All the healthcare workers (HCWs) were tested on day 0 (before the first dose of vaccine; T0) for IgG against the nucleocapsid (N) protein and Spike RBD IgG against SARS-CoV-2. The antibody against N protein was taken as a proxy for previous SARS-CoV-2 infection (7). Subsequently, the samples were collected after the 4th (before the second dose of vaccine), 8th, 12th, 16th, 20th, and 24th week of the first dose of vaccine (noted as T1, T2, T3, T4, T5, and T6, respectively).

### Participant Enrollment and Data Collection

We invited for voluntary participation of the HCWs in the study from December 15, 2020 to January 15, 2021 through a letter published by all the 6 participating institutions in this study and we have ended up with the enrollment of 614 HCWs in our cohort based on the inclusion criteria. Participants were explained about this study and a written informed consent was obtained from each participant prior to enrollment. Inclusion criteria included the following: (a) Participant should be adult, (b) willing to get vaccinated against SARS-CoV-2, (c) ready to give a blood sample at each point of time, (d) signed a written informed consent, and (e) willing to share the required information. We have followed these enrolled participants up to the mentioned timeline to collect blood samples and required information at each timepoints. All the participants were interviewed for demographic characteristics, SARS-CoV-2 infection history, vaccination details, and the information was recorded in the ODK app (an electronic-based open data collection kit).

### Test Method

A serum sample from each participant was used to detect the immunoglobulin G (IgG) antibodies against N and S protein antigen using commercially available chemiluminescent microparticle immunoassay (CLIA). Total antibodies (including IgG) against the nucleocapsid protein were estimated in Roche Cobas e411 (Roche Diagnostics Int. Ltd., Mannheim, Germany) using an *in-vitro* qualitative kit Elecsys^®^ Anti-SARS-CoV-2 as per the instructions of the manufacturer. Spike RBD IgG antibodies against SARS-CoV-2 were estimated in ARCHITECT i1000SR (Abbott Diagnostics, Chicago, USA) using a commercial quantitative kit ARCH SARS-CoV-2 IgG II Quant (Abbott Diagnostics, Chicago, USA) according to the instructions of the manufacturer. A cutoff index (COI) of ≥ 1.0 was interpreted as reactive and < 1.0 as non-reactive for Elecsys^®^ Anti-SARS-CoV-2. The cutoff value for the quantitative kit was 50 AU/ml.

### Statistical Analysis

Descriptive statistical analyses were performed using the GraphPad Prism 9.00 for Windows (GraphPad Software, La Jolla, California, USA) and the SPSS software (IBM SPSS Statistics for Windows, version 24.0, Armonk, New York, USA). The statistical significance threshold was set at 5%.

### Study Approval

This study was ethically approved by the Institutional Human Ethical Committee of ICMR–Regional Medical Research Center, Bhubaneswar vide no ICMR-RMRCB/IHEC-2020/036 dated 07/11/2020.

## Results and Discussion

Among the 614 participants enrolled in the study, 308 (50.2%) participants were recipients of the Covishield vaccine and the rest 306 (49.8%) participants had received Covaxin. The participants included 396 (64.5%) males and 218 (35.5%) females. The median age of all the participants was calculated as 37 years [interquartile (IQ): 28–47 years]. A total of 42.4% (260 of 614) participants had the previous history of SARS-CoV-2 infection prior to administration of either vaccine and the median antibody titer against N-protein among them was 19.70 COI (IQ: 6.74–76.2).

**Figure 1:**
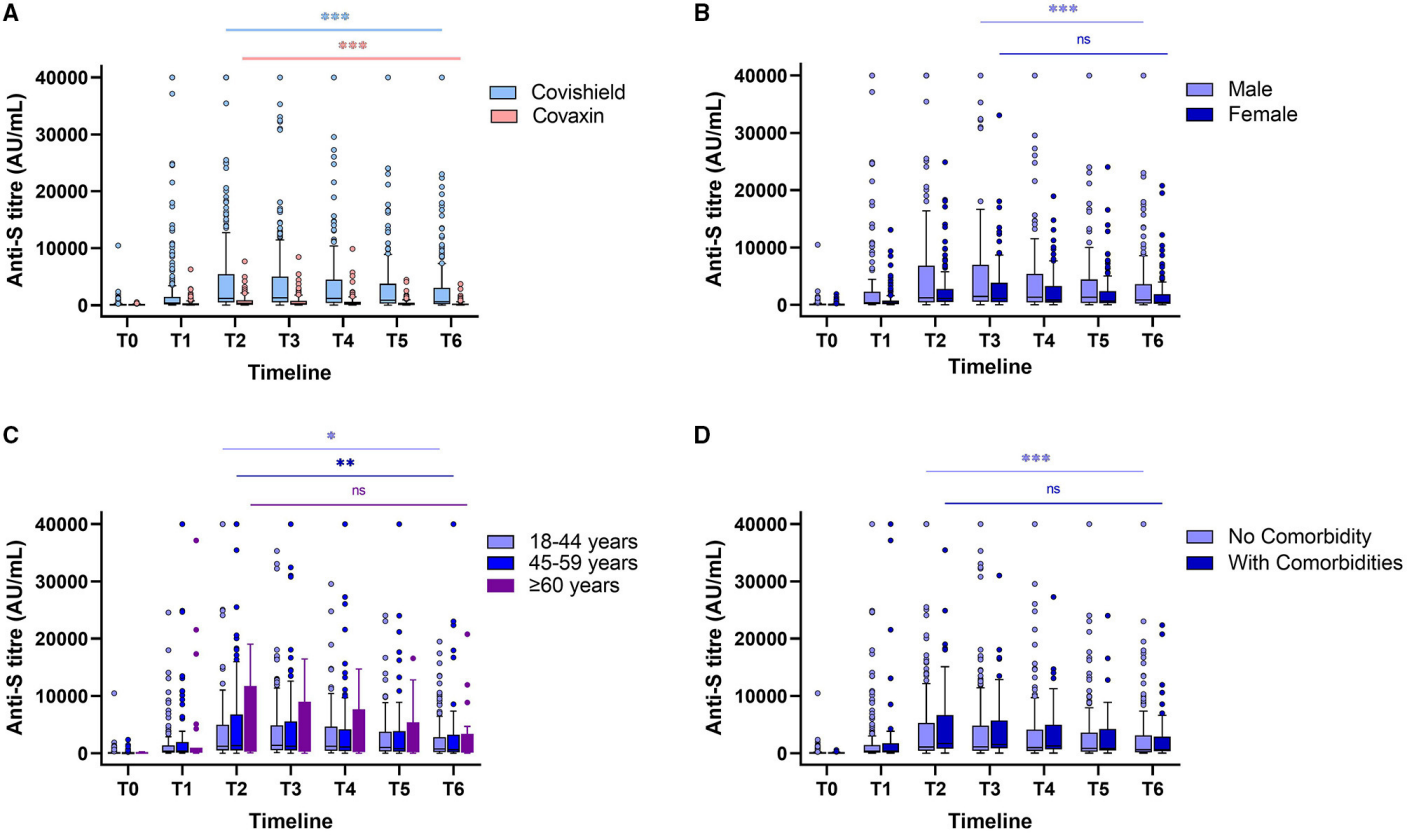
Levels of antibody against spike (S) glycoprotein at different timepoints after a complete dose of Covishield and Covaxin recipient without any breakthrough infection **(A)**. Anti-S antibody levels of Covishield recipients stratified by gender **(B)**, age **(C)**, and comorbidities **(D)**. “ns” indicates nonsignificant; ^*^indicates *p* < 0.05; ^**^indicates *p* < 0.01; ^***^indicates *p* < 0.001. The Tukey's method was used to plot whiskers.

**Figure 2:**
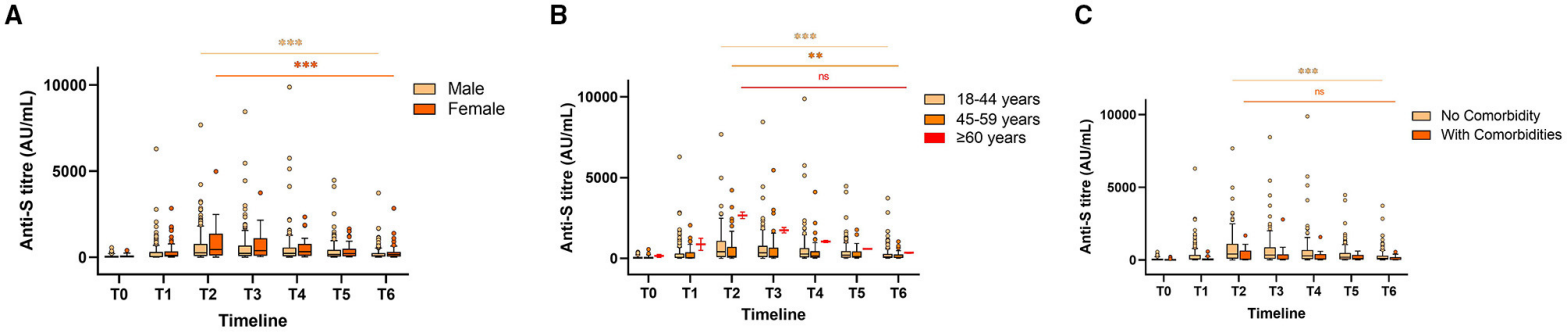
Anti-S antibody levels stratified by gender **(A)**, age **(B)**, and comorbidities **(C)** in Covaxin recipients at different timepoints. “ns” indicates non-significant; ^**^indicates *p* < 0.01; ^***^indicates *p* < 0.001. The Tukey's method was used to plot whiskers.

The results of 533 (86.8%) HCWs without any postvaccination history of infection indicated a drop of almost 2- to 3-fold in the Spike RBD IgG median concentration for both the vaccines. There was no statistically significant difference in postvaccination antibody production and its persistence across gender, age, comorbidities, and blood groups (Table 1, Figures 1, 2). The lowest antibody median level (141.6 AU/ml; IQ: 99.0–1,502.8) and maximum waning (4-fold) was observed among the participants with A-blood group after a complete dose of either vaccine (Table 1). The production of vaccine-induced IgG antibodies was significantly higher (*p* < 0.001) in Covishield recipients compared with Covaxin recipients. Covishield recipients produced the highest median of 1,299.5 AU/ml (IQ: 517.9–5,019.07) for anti-S IgG which is almost 4-fold higher than the Covaxin-induced antibody concentration of 342.7 AU/ml (IQ: 76.1–892.8) (Figure 1). In seronegative individuals, the rate of seroconversion after 28 days of the first dose was 81.9% for Covishield (Figure 1) and 16.1% for Covaxin (Figure 2). The highest antibody level for Covaxin recipients was recorded at T2 that started to decrease from T3 (median = 305.18 AU/ml; IQ: 78.2–771.2) and was recorded as 95.1 AU/ml (IQ: 36.5–277.2) after a 3-fold drop at T6. The maximum median of anti-S IgG for the Covishield recipient was recorded at T3 and started to decrease significantly (*p* = 0.001) from T4 (median=1,177.3 AU/ml; IQ: 412.4–4,526.5). The lowest antibody level was noted at 637.2 AU/ml (IQ: 186.5–3,055.3) after 6 months from the first vaccine shot (*p* < 0.001).

**Table 1:** Demographic data of included participants without breakthrough infection.

**Variables**		**N (%)**	**T0**		**T1**		**T2**		**T3**		**T4**		**T5**		**T6**	
			**Median** **(Q1 – Q3)**	***p*** **-value**	**Median** **(Q1 – Q3)**	***p*** **-value**	**Median** **(Q1 – Q3)**	***p*** **-value**	**Median** **(Q1 – Q3)**	***p*** **-value**	**Median** **(Q1 – Q3)**	***p*** **-value**	**Median** **(Q1 – Q3)**	***p*** **-value**	**Median** **(Q1 – Q3)**	***p*** **-value**
Gender	Male	341 (64.0)	9.4 (3.6–99.0)	0.600	212.3 (37.5–721.7)	0.250	640.3 (140.8–1949.7)	0.172	566.7 (135.9–1755.9)	0.113	448.9 (119.4–1465.2)	0.141	342.4 (99.3–1331.9)	0.111	217.2 (60.6–1023.4)	0.076
	Female	192 (36.0)	9.5 (3.2–93.8)		257.9 (53.7–663.6)		959.2 (208.1–1987.4)		763.0 (247.0–1738.9)		570.2 (231.7–1723.4)		438.9 (196.6–1284.3)		284.7 (110.9–813.3)	
Age Groups	18–44 Years	356 (66.8)	9.7 (3.6–99.0)	0.401	213.7 (45.3–578.8)	0.075	662.55 (158.1–1834.3)	0.217	567.5 (169.825–1555.475)	0.160	478.6 (151.675–1304.225)	0.303	369.35 (103.025–1161.9)	0.260	227.45 (68.6325–813.025)	0.119
	45–59 Years	152 (28.5)	9.7 (3.4–89.6)		253.3 (42.0–797.2)		829.60 (135.6–2436.4)		780.3 (146.2–2198.275)		528.15 (138–2277.925)		439.3 (111.925–1782.8)		288.5 (75.75–1306.15)	
	Above 60 Years	25 (4.7)	4.8 (2.5–111.4)		374.2 (102.4–1117.7)		978.30 (303.3–7348.3)		744.62 (311.65–6781.3)		504.6 (224.4–4858.6)		315.7 (209–3657.2)		326.6(145.8–2612.4)	
Blood Groups	A+	103 (19.3)	7.8 (3.34–99.07)	0.533	137.2 (34.2–478.4)	0.244	557.8 (100.5–1471.5)	0.092	504.6 (89.41–1554.2)	0.105	389.4 (75.6–1165.6)	0.116	298.2 (71.5–854.3)	0.174	179.4 (50.6–656.71)	0.157
	B+	198 (37.1)	15.8 (4.5–95.5)		264.5 (54.2–761.2)		706.05 (263.575–2563.25)		667.7 (233.975–2204.505)		582.85 (208.35–1953.075)		419.455 (159.375–1582.875)		290.65 (107.55–983.425)	
	AB+	42 (7.9)	18.0 (3.2–86.5)		181.6 (34.8–416.0)		739.05 (230.245–1507.05)		597.35 (249.925–1568.05)		487.65 (207.575–1329.725)		417 (158.8–1290.175)		243.35 (81.025–1209.95)	
	O+	180 (33.8)	8.7 (2.7–99.4)		229.0 (45.7–857.1)		718.9 (137.315–1854.6)		662.7 (137.2–1650.625)		507.8 (127.975–1638.525)		428.3 (101.1–1419.275)		244.55 (71.4–1064.25)	
	A–	3 (0.6)	16.7 (10.4–131.1)		79.3 (52.4–662.1)		141.6 (99–1502.8)		92.8 (70.6–1005.4)		80.7 (61.7–598.5)		48.4 (42.6–324.2)		34.7 (32.5–176.9)	
	B–	4 (0.8)	84.4 (16.2–187.6)		278.6 (80.5–802.1)		994.3 (240.125–1624.8)		737.5 (201.6–1360.1)		602.805 (163.9075–1242.2525)		522.655 (142.8–1085.1275)		419.8 (116.8575–863.975)	
	O–	3 (0.6)	89.7 (49.3–95.5)		740.7 (470.2–797.5)		2004.31 (1753.7–2452.8)		1743.5 (1450.6–3297)		1474.2 (1104.2–2371.7)		1191.4 (813.6–1655)		840.6 (545.1–1151.8)	
Comorb–idities	Present	77 (14.4)	6.9 (2.5–93.6)	0.070	223.8 (29.3–578.3)	0.682	938.2 (86.0–2537.0)	0.579	921.4 (159.4–2627.7)	0.393	776.8 (182.3–2028.4)	0.283	567.8 (139.2–1897.4)	0.147	400.7 (79.7–1606.0)	0.151
	Absent	456 (85.6)	10.6 (3.7–98.3)		227.0 (46.0–723.6)		664.5 (158.1–1905.5)		602.3 (179.1–1630.7)		480.8 (156.6–1453.6)		360.8 (112.5–1240.7)		231.0 (75.7–849.3)	
Vaccine	Covaxin	255 (47.8)	9.2 (3.5–94.6)	0.503	61.4 (22.0–321.5)	<0.001^*^	342.7 (76.1–892.8)	<0.001^*^	305.2 (78.2–771.2)	<0.001^*^	249.8 (63.0–637.1)	<0.001^*^	179.1 (47.3–451.6)	<0.001^*^	95.1 (36.5–277.1)	<0.001^*^
	Covishield	278 (52.2)	9.7 (3.4–100.8)		365.3 (132.7–1483.1)		1223.3 (482.2–5476.0)		1299.5 (517.9–5019.2)		1177.3 (412.4–4526.5)		888.4 (300.8–3785.0)		637.2 (186.5–3055.3)	

* *denotes statistically significant*.

Among the 224 (36.5%) seropositive cases without reinfection, the median antibody titer for Covaxin was 102.7 AU/ml (IQ: 75.2–154.4) and for Covishield, it was 125.3 AU/ml (IQ: 80.5–339.5) at T0. The highest median antibody titer was recorded at 884.7 AU/ml (IQ: 579.4–1,795.5) and 6,286 AU/ml (IQ: 2,307.1–12,126.5) in T2 for Covaxin and Covishield, respectively. Vaccine-induced antibodies were observed to be declined with time and the level waned to 276.8 AU/ml (IQ: 179.6–471.9) and 2,813.6 AU/ml (IQ: 1,417.9–5,112.5) at T6 for Covaxin and Covishield recipients, respectively. The median antibody titer for reinfection cases in T0 was 75.4 AU/ml (IQ: 66.8–99.9) for Covaxin and 78.8 AU/ml (IQ: 62.7–125.9) for Covishield.

**Figure 3:**
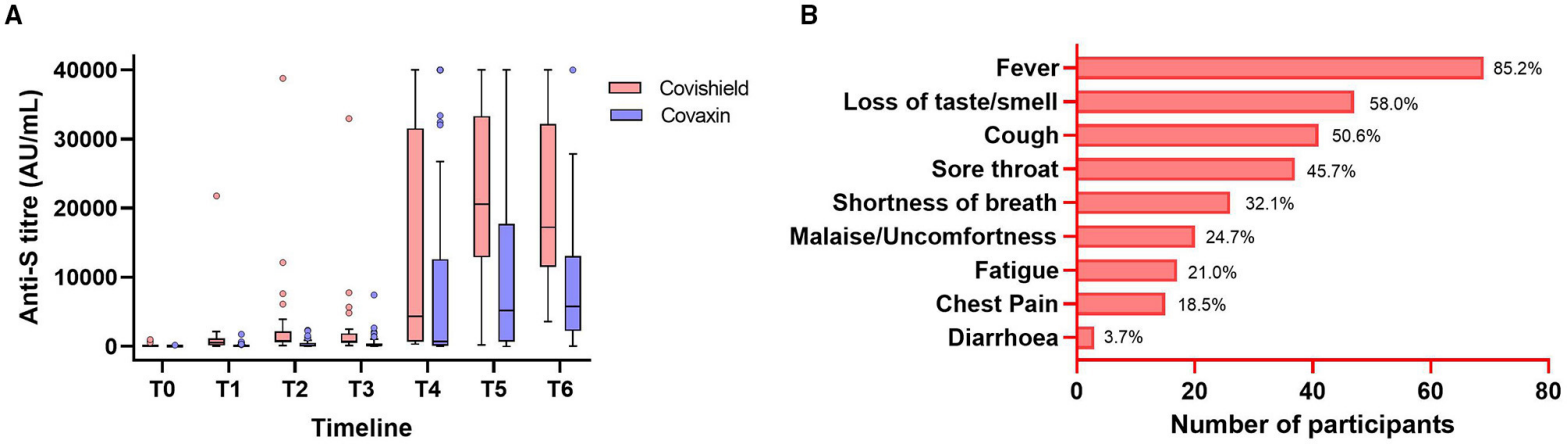
Anti-S immunoglobulin G (IgG) antibody levels at different timepoints of the Covishield and Covaxin vaccine recipients having breakthrough infection **(A)**. The symptomatic status with percentage of all the breakthrough cases **(B)**. The Tukey's method was used to plot whiskers.

In this study, 13% (81 of 614) HCWs were reported for breakthrough infection, and the antibody production and persistence among them were separately analyzed. Within those breakthrough cases, 63% (51 of 81) were Covaxin recipients, 37% [30] were Covishield recipients, 55.6% [45] had no previous history of SARS-CoV-2 infection, and 44.4% [36] of HCWs were having previous infection history. The Spike RBD IgG titer of 81 breakthrough cases was recorded at 345.6 AU/ml (IQ: 62.9–879.2) in T2, which increased further to 10,550.4 AU/ml (IQ: 3,635–21,803.9) at T6 (Figure 3). However, the median antibody titer level was noted as 269.1 AU/ml (IQ: 51.5–740.7) in individuals immediately prior to breakthrough infection. Among those, 75 (92.5%) had mild symptoms and 9 (11.1%) participants were hospitalized. Fever was the primary symptom in 85.2% of individuals followed by loss of taste/smell (58.0%), cough (50.6%), sore throat (45.7%), and shortness of breath (32.1%) among the other significant symptoms (Figure 3). Among 36 reinfection cases, an equal number of participants were administered with Covaxin and Covishield. The median antibody titer in those reinfection cases was 305.45 AU/ml (IQ: 197.48–864.1) among Covaxin recipients and 723.95 AU/ml (IQ: 412.45–2,826.3) in Covishield recipients. The median days of reinfection with COVID-19 were observed as 188 days.

This study reports a significant decrease in antibody level after 2 months among Covaxin and 4 months among Covishield recipients post double doses of the BBV-152 and AZD1222 vaccines. The highest median antibody titer was observed after 4 weeks of double dose for all the seropositive (at T0) participants. The antibody titer increased ~ 17-fold in those participants when compared with their antibody level at T0 and is in corroboration to other reports from elsewhere (14, 15). Nearly half of the breakthrough cases were found in HCWs were previously infected at different timepoints. This may be because of the waning the naturally developed antibody or due to the emergence of new SARS-CoV-2 variants (16).

Protection imparted by natural SARS-CoV-2 infection, the threshold required for giving protective immunity and persistence of the antibody is yet to be understood completely (17, 18). One of the limitations of this study could be the unavailability of a neutralization test. However, the determination of anti-S IgG titer is more practical as neutralizing antibody titers are typically not readily available (19). A significant correlation has also been reported between neutralizing antibody titers and anti-S or antireceptor binding domain IgG antibody titers (20). Participation of only HCWs could be another limitation of this study which restricts the generalization of the findings.

To the best of our knowledge, this study reports for the first time about the 6 months persistence of spike RBD antibody after Covaxin (BBV-152) vaccination although limited data exists on long-term antibody kinetics among the AZD1222 vaccine recipients. Various studies carried out with BNT162b2 (Pfizer–BioNTech) and ChAdOx1 nCoV-19 (Oxford–AstraZeneca) have shown a 2-fold decrease for BNT162b2 between 21 and 41 days and 5-fold drop in ChAdOx1 after 70 days of complete vaccination (7, 21).

The 6 months follow-up study documents a 2-fold and 4-fold decrease in antibody titer among the Covishield and Covaxin recipients, respectively. The clinical implications of the decline in vaccine-induced antibodies are yet to explain properly, and it remains crucial to establish S-antibody thresholds associated with protection against clinical outcomes. Emerging evidence suggests that antibodies are particularly important for restricting infection and preventing the transmission of the virus, whereas T cells may be particularly relevant for preventing severe disease and death. Findings from this study suggest for a larger study that would help to define correlates of protection and generate substantial data to determine whether there is a need to produce modified vaccines, or booster doses. This study has a follow-up plan for two years which will further help in understanding the kinetics model and also to provide a better estimate of the antibody response in both the seropositive and seronegative individuals over a significant period.

## Data Availability Statement

The original contributions presented in the study are included in the article/Supplementary Material, further inquiries can be directed to the corresponding author/s.

## Ethics Statement

The studies involving human participants were reviewed and approved by the Institutional Human Ethical Committee of ICMR – Regional Medical Research Centre, Bhubaneswar vide no ICMR-RMRCB/IHEC-2020/036 dated 07/11/2020. The patients/participants provided their written informed consent to participate in this study.

## Author Contributions

DB and SPa conceptualized the study. JK, HC, PC, KP, NR, SPo, AM, SKPr, MP, and RN collected blood samples, information from participants, and written informed consents. DPar, SS, and UR performed the laboratory tests. DPar, GC, and HC have done the data analysis. HC, DPar, and DB drafted the original manuscript. Manuscript review and editing were done by NMi, DPat, SB, SR, and SM. NMo, SK, SKPa, DB, and SPa supervised the study. All the authors have read and reviewed the manuscript and gave their final approval.

## Conflict of Interest

The authors declare that the research was conducted in the absence of any commercial or financial relationships that could be construed as a potential conflict of interest.

## Publisher's Note

All claims expressed in this article are solely those of the authors and do not necessarily represent those of their affiliated organizations, or those of the publisher, the editors and the reviewers. Any product that may be evaluated in this article, or claim that may be made by its manufacturer, is not guaranteed or endorsed by the publisher.
