# Supplementary material for: Persistence of Antibodies Against Spike Glycoprotein of SARS-CoV-2 in Healthcare Workers Post Double Dose of BBV-152 and AZD1222 Vaccines
Source: Front Med (Lausanne). 2021 Dec 22;8:778129. doi: 10.3389/fmed.2021.778129 (PMC8727751; doi:10.3389/fmed.2021.778129)
Supplement: Supplementary file 1 [file Table_1.DOCX]

**Cohort Follow up questionnaire**

1. Site Name:
2. Name:
3. Sample ID:
4. Age:
5. Sex:
6. Previous history of COVID-19: Yes / No
7. If yes, date of infection:
8. Which vaccine you have taken?
9. Date of Vaccination (1st Dose):
10. Date of Vaccination (2nd Dose):
11. Blood group:
12. Any chronic disease present:
    - 1. High blood pressure
      2. Blood sugar
      3. Chronic Kidney Disease
      4. Chronic obstructive pulmonary disease
      5. Chronic Heart disease
      6. Liver disease
      7. Cancer
13. Infection post vaccination: Yes / No
14. Hospitalization: Yes / No
15. Any symptoms present:
    - 1. Fever
      2. Loss of taste/smell
      3. Shortness of breath
      4. Chest Pain
      5. Sore throat
      6. Fatigue
      7. Malaise/Uncomfortness
      8. Diarrhoea
      9. Cough
